# Giant renal schwannoma with obvious hemorrhage and cystic degeneration: a case report and literature review

**DOI:** 10.1186/s12894-022-01058-9

**Published:** 2022-07-11

**Authors:** Chao Feng Yang, Hui Zuo, Jin Hong Yu, Sushant Kumar Das, Yang Li

**Affiliations:** 1 Sichuan Key Laboratory of Medical Imaging, Department of Radiology, The Affiliated Hospital of North Sichuan Medical College, 63 Wenhua Road, Nanchong City, 637000 Sichuan Province China; 2 Sichuan Key Laboratory of Medical Imaging, Department of Ultrasound, The Affiliated Hospital of North Sichuan Medical College, 63 Wenhua Road, Nanchong City, 637000 Sichuan Province China; 3 Department of Ultrasound, The People’s Hospital of Yuechi County, 22 East Jianshe Road, Yuechi County, 638350 Sichuan Province China; 4 Department of Radiology, The People’s Hospital of Yuechi County, 22 East Jianshe Road, Yuechi County, 638350 Sichuan Province China

**Keywords:** Renal tumor, Renal schwannoma, Computed tomography, Magnetic resonance imaging

## Abstract

**Background:**

Renal schwannomas are very rare and are usually benign. Its clinical symptoms and imaging features are nonspecific, and the diagnosis is usually confirmed by pathology after surgical resection.

**Case presentation:**

A 46-year-old Chinese female was admitted to the hospital with right flank pain that had persisted for the six months prior to admission. This pain had worsened for 10 days before admission, and dyspnea occurred when she was supine and agitated. A right abdominal mass could be palpated on physical examination. Computed tomography and magnetic resonance imaging examinations revealed a large, nonenhanced, cystic and solid mass in the right kidney. The patient received radical nephrectomy for the right kidney. The diagnosis of schwannoma was confirmed by pathological examination.

**Conclusions:**

We report a case of a large renal schwannoma with obvious hemorrhage and cystic degeneration, which can be used as a reference for further study.

## Background

Schwannomas are predominantly benign peripheral nerve sheath tumors. These tumors rarely undergo malignant transformation. Schwannomas are most commonly seen in the extremities, head, neck, retroperitoneum and mediastinum. Rarer locations include the kidney, duodenum [1], bronchus [2], and other internal regions. Renal schwannomas more frequently arise from the hilum and less frequently arise from the parenchyma because nerve tissues congregate at the hilum [3]. We report a case of large schwannoma originating from the renal parenchyma.

## Case presentation

**Fig. 1:**
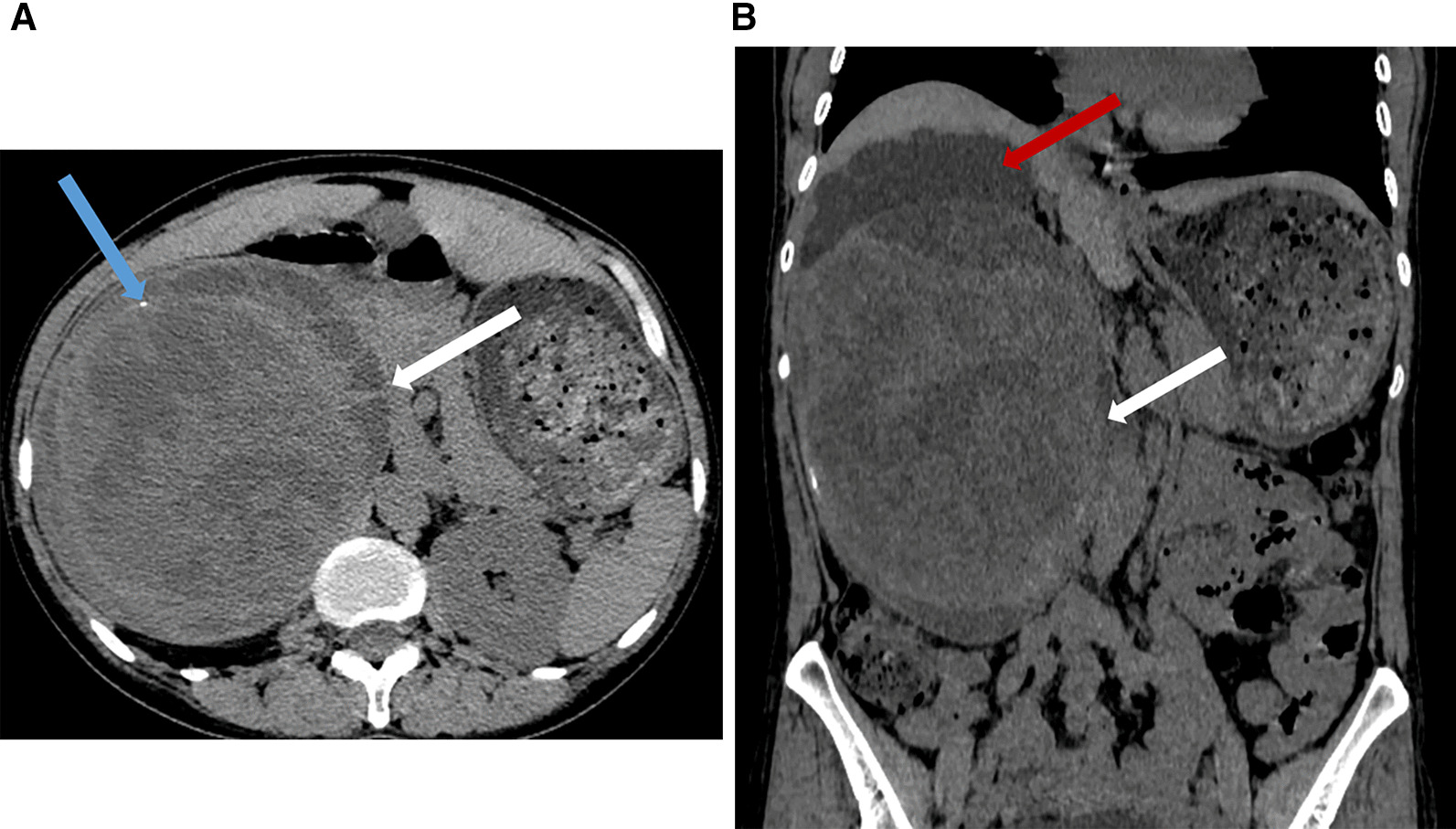
CT findings of renal schwannoma. Axial (**a**) and coronal (**b**) CT scan shows a giant cystic and solid mass (white arrow) is located at right kidney with a few calcification (blue arrow) and hydronephrosis (red arrow)

**Fig. 2:**
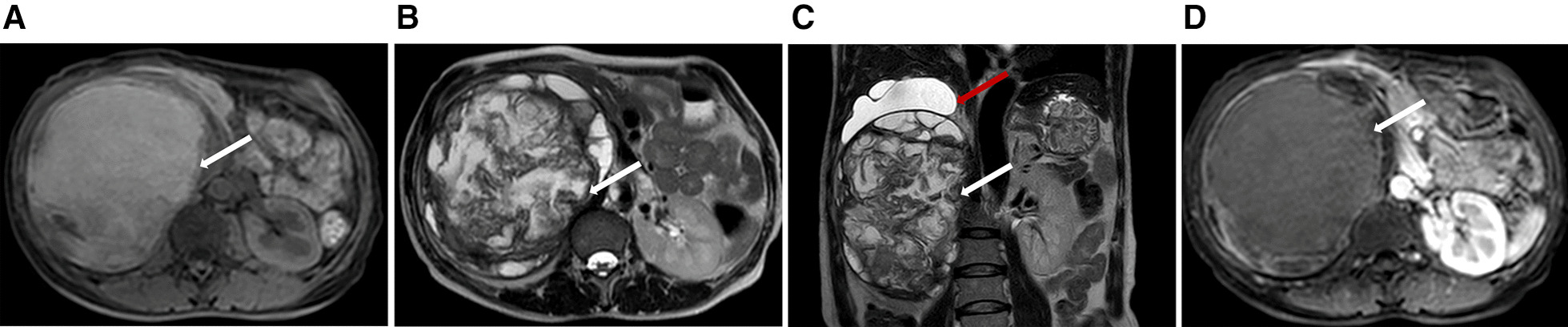
MR findings of renal schwannoma. **a** Precontrast T1-weighted MR shows a slightly hyperintense mass (white arrow) arises from the kidney. T2-weighted axial (**b**) and cronal (**c**) plane MR shows the high-low mixed signal intensity mass (white arrow) with hydronephrosis (red arrow). No obvious enhancement (white arrow) on enhanced T1-weighted imaging (**d**)

**Fig. 3:**
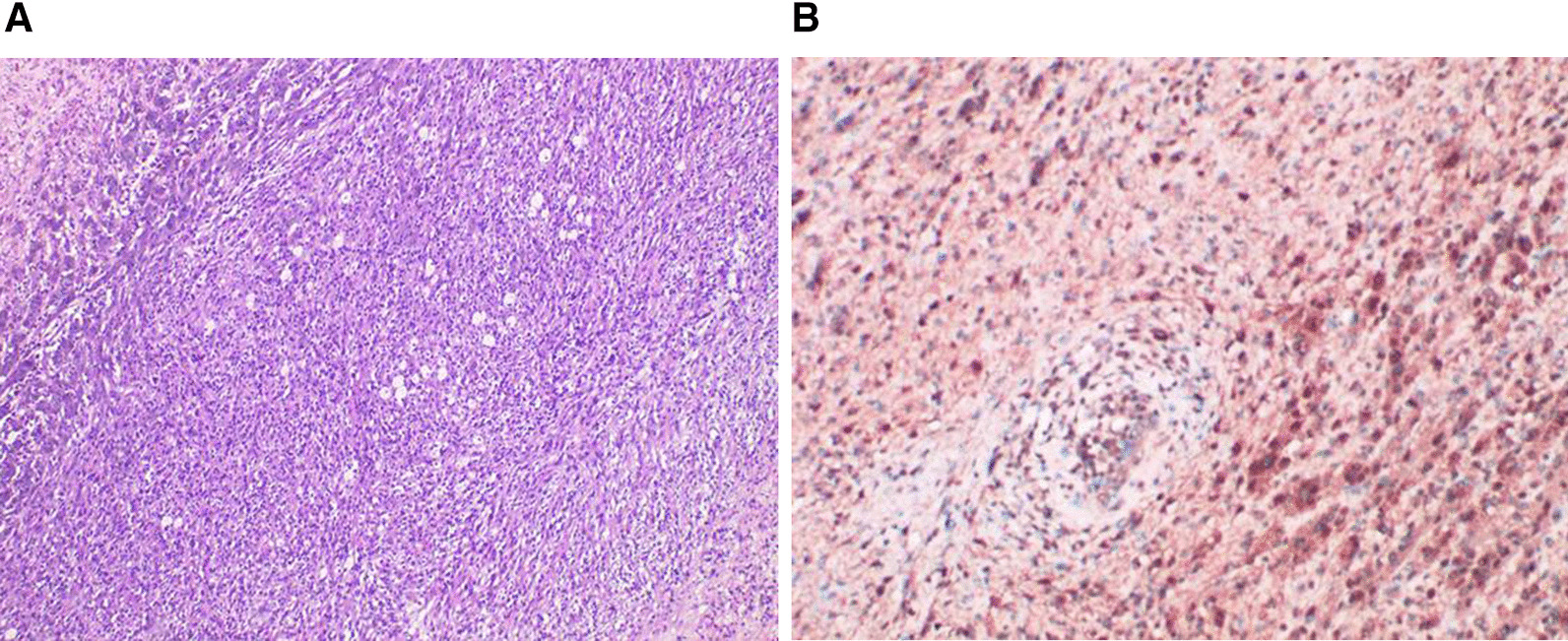
Final pathology slide of nephrectomy specimen. **a** H&E staining showed the tumor was fairly cellular showing spindle cells (× 100). **b** Immunostaining with S-100 protein was positive (× 100)

A 46-year-old Chinese female had right flank of unclear origin pain that lasted more than six months. It began with slight and persistent dull pain and no other symptoms. Ten days before admission, however, the pain worsened, and dyspnea occurred when she was supine and agitated. A right abdominal mass with poor mobility and a clear boundary between the surrounding structures could be palpated on physical examination. There was no tenderness. The patient had no genetic history of neurofibromatosis. No abnormal findings were found on blood, biochemistry, routine urine and antibody laboratory examination. Nonenhanced CT showed a large cystic and solid mass in the right kidney with septation and a few areas of calcification that increased the volume of the right kidney. The renal cortex had become thinner, and the renal pelvis and calices were obviously hydrous and dilated. The adjacent organs were compressed and displaced (Fig. 1). MRI revealed that the mass was slightly hyperintense on T1-weighted imaging, and had high-low mixed signal intensity on T2-weighted imaging. The edge of the lesion showed hyperintensity on diffusion-weighted imaging, and ring-like and septal enhancement was observed on enhanced T1-weighted imaging (Fig. 2). Because the mass was so large, the patient underwent radical nephrectomy of the right kidney, which revealed that the mass had adhered tightly to the inferior vena cava and duodenum. Postoperative pathology showed that the mass from the renal parenchyma measured 20.5 × 17.5 × 10.0 cm and was encapsulated. On cut sections, it was soft and reddish-brown with a massive amount of hemorrhage and necrosis. The boundary between the mass and renal parenchyma was clear. Immunostaining with S-100 protein and Ki-67 (positivity in approximately five percent of neoplastic cells) was positive and supported a diagnosis of a benign schwannoma (Fig. 3). Postoperatively, the patient recovered well, and no complications were observed.

## Discussion and conclusions

Peripheral schwannoma is an uncommon tumor that originates from Schwann cells of nerve sheaths. Approximately three percent of schwannomas occur retroperitoneally, but renal involvement is uncommon [4]. Most of them are benign, and malignancy is rare. There are different types of schwannomas: plexiform, ancient, cellular, melanotic, epithelioid, and microcystic [5]. In the 2020 WHO Classification of Tumors of Soft Tissue, melanotic schwannoma was reclassified as a malignant tumor because of its aggressive clinical behavior [6].

There are only 37 cases of renal schwannoma reported in the English literature. Table 1 summarizes the data for these cases, including the present case. Among the 37 cases, the mean age of the patients was 52.0 ± 14.0 years (range 18–74 years), and the male to female ratio was 1:1.5. The mean size of the lesions was 8.0 ± 4.3 cm (range 2.6–20.5 cm). The renal schwannomas were all solitary. There were 16 lesions in the left kidney and 21 lesions in the right kidney, and the ratio of left to right was approximately 1:1.3. These lesions were located at the hilum and pelvis (51.4%), parenchyma (43.2%) and capsule (5.4%). Among all cases, 33 were benign, and 4 were malignant. Malignant renal schwannoma can metastasize to the lung, bone, diaphragm, liver, colon, mesentery, peritoneum, and subcutaneous tissues, of which lung metastasis is the most common.

**Table 1:** Cases of renal schwannoma

Author	Year	Sex	Age (years)	Side	Location	Size (cm)	Malignancy	Symptom	Imaging features
Phillips [7]	1955	M	56	L	Hilum	12	No	Fever, chills, weight loss	A large, diffuse, smooth shadow on excretory and retrograde pyelography
Fein [8]	1965	F	51	R	Hilum	6	No	Fever, chills, right flank pain, dysuria	Renal hypertrophy, pyelectasis and caliectasis on retrograde pyelography
Bair [9]	1978	M	56	R	Hilum	7	No	Microscopic hematuria	Neovascularity within a solid mass on selective right renal arteriography
Steers [10]	1985	F	50	R	Hilum	9	No	Microscopic hematuria	A noncalcified, cystic renal mass with hemorrhage and nerosis on CT; hypovascular exophytic mass on renal arteriography
Somers [11]	1988	F	55	L	Parenchyma	5	No	Incidental finding	Solid mass on arteriography
Kitagawa [12]	1990	M	51	L	Hilum	2.8	No	Epigastric pain, high fever	Hypoechoic mass on US; an extrinsic compression of the left renal pelvis and mild hydronephrosis on excretory pyelography; homogeneous tumor without enhanced on CT; isointense on T1WI, homogeneous hyperintense on T2WI
Ma [13]	1990	M	67	R	Parenchyma	8	No	Epigastric pain	Renal hypertrophy on US; hypovascular tumor on arteriography
Naslund [14]	1991	F	50	L	Parenchyma	14	Yes	Mild abdominal discomfort,decreased appetite, weight loss	–
Romics [15]	1992	M	52	R	Capsule	A large invasve mass	Yes	Back and the right flank pain, recurrent fever	Extensive, cystic-necrotic space occupation in the right kidney on imaging techniques
Singer [16]	1996	F	70	L	Hilum	6	No	Asymptomatic	Extrinsic compression of the upper and middle infundibula on excretory pyelography; soft tissue mass with moderate enhancement on CT; slightly hypointense on T1WI, slightly hyperintense on T2WI
Alvarado-Cabrero [17]	2000	F	18	R	Parenchyma	6.2	No	Flank pain	–
Alvarado-Cabrero [17]	2000	F	40	L	Parenchyma	12.5	No	Flank pain	–
Alvarado-Cabrero [17]	2000	M	45	L	Parenchyma	16	No	Flank and abdominal pain	–
Tsurusaki [18]	2001	F	69	L	Capsule	–	No	Incidental finding	Severe extrinstic compression of the left ureter on excretory pyelography; heterogeneous hypoechoic mass on US; low-attenuation area with moderately enhanced rim on CT; hypointense on T1WI, slightly hyperintense on T2WI
Cachay [19]	2003	F	74	R	Parenchyma	9	Yes	–	An unique, well-demarcated, round hypodense mass on CT
Singh [20]	2005	M	40	L	Pelvis	3	No	Left renal colicky pain	A soft tissue mass on US; enhancing mass compresses the pelvis of the left kidney on CT
Singh [20]	2005	M	35	R	Hilum	–	No	Fank pain, hematuria	A heterogenous mass on US; enhancing mass compresses the renal parenchyma and pelvis on CT
Umphrey [21]	2007	F	63	R	Parenchyma	7	No	Hypertension and hot flashes	A markedly hypoechoic mass with lobulation and septations on US; a lower attenuation large lobulated mass with a few faint calcifications and slight enhancement on CT
Hung [22]	2007	F	36	L	Parenchyma	7.3	No	Low-grade fever	Homogeneous isointense on T1WI, homogeneous hyperintense on T2WI, enhancement of outer rim of tumor on gadolinium-enhanced T1WI
Gobbo [23]	2008	F	59	L	Hilum	4.8	No	Asymptomatic	–
Gobbo [23]	2008	F	27	R	Parenchyma	8.5	No	Incidental finding	–
Gobbo [23]	2008	F	35	L	Hilum	7	No	Abdominal pain, nausea	–
Chen [24]	2010	M	34	R	Hilum	2.6	No	Hematuria	Solid mass on US; the edge of right renal calices was irregular on excretory pyelography; solid mass with slight enhancement on CT
Nayyar [25]	2011	F	44	R	Hilum	10	No	Flank pain, nausea, vomiting	A large cystic area with large extrarenal pelvis and gross hydronephrosis on US, CT and excretory pyelography
Yang [26]	2012	F	40	L	Pelvic	6.8	No	Flank pain	A low-attenuated, lobulated, and minimally enhanced on CT; on retrograde pyelography, the left ureteropelvic junction was kinked, the upper calyces were obliterated, and the calyx was filled with an irregular collection of contrast
Wang [27]	2013	M	66	L	Parenchyma	2.7	No	Intermittent painless gross hematuria	A solid mass on CT
Mikkilineni [28]	2013	F	36	R	Parenchyma	4.6	No	Fever, malaise, right flank discomfort, nignt sweat, hematuria	A complex cystic leision on US; a complex cystic leision with thick, irregular, nodular rim of enhancement on CT
Verze [3]	2014	M	59	R	Parenchyma	15	Yes	Incidental finding	A mass with a large central necrosis on CT
Yong [29]	2015	F	55	R	Pelvic	5.1	No	Colicky pain, microscopic haematuria	A soft tissue density lesion with mildly enhancement on CT;
Hall [30]	2015	F	73	L	Hilum	4.9	No	Vague abdominal pain	An echo poor mass on US; smooth filling defect affecting the renal pelvic on retrograde pyelography
Kumano [31]	2015	M	73	R	Hilum	3.5	No	–	The tumor was poorly enhanced on CT; MRI showed that the inside was uniform on T1WI and heterogeneous contrast on T2WI
Herden [32]	2015	M	60	R	Parenchyma	11	No	Incidental finding, asymptomatic	A polycystic, centrally hypodense space-occupying mass with rim of enhancement on CT
Herden [32]	2015	F	69	R	Hilum	6.5	No	Microscopic hematuria	A tumorous space-occupying process with partially central colliquations, compressing the vena cava on CT
Vidal [33]	2020	M	66	R	Parenchyma	3.5	No	Incidental finding	A focal solid mass on US and CT
Wang [34]	2020	F	56	L	Hilum	11.5	No	Left lower back pain	A massive tumor with soft tissue density and inhomogeneous enhancement on CT
Dahmen [35]	2021	M	47	R	Hilum	12	No	Flank pain	Enhancing large right renal mass with no filling defect of the renal pelvis on CTU
Present	2021	F	46	R	Parenchyma	20.5	No	Flank pain	A cystic and solid mass with septation and local calcification on CT; slightly hyperintense on T1WI, high-low mixed signal intensity on T2WI, ringlike and septal enhancement

We further analyzed the radiological images of these previously reported renal schwannomas and summarized some of its radiological features. On ultrasound (US), renal schwannomas are hypoechoic, well-defined masses and may contain cystic areas, which are more commonly seen in renal schwannomas, as they are larger than 5 cm. The larger the tumor is, the more cystic degeneration and necrosis are present. Further investigation is usually performed with CT or MR imaging. On nonenhanced CT, they are typically well-defined and round or fusiform hypoattenuating masses. Large tumors also show cystic degeneration, calcifications and hemorrhage. On contrast-enhanced CT, renal schwannomas show mild to moderate homogeneous or heterogeneous enhancement. On MR imaging, most renal schwannomas often appear isointense or hypointense relative to muscle on T1-weighted imaging and hyperintense on T2-weighted imaging with variable enhancement; however, cystic degeneration and hemorrhage can complicate the signal intensity. In addition, pyelectasis and caliectasis on excretory and retrograde pyelography and hypovascular tumors on renal arteriography can be seen. There are no clear imaging features that can differentiate between benign and malignant lesions unless metastasis in other regions is found.

There are two important differences between the present case and the previous cases. First, the patient in question here had a large renal schwannoma, which is quite rare, making it the largest tumor reported thus far. Second, to the best of our knowledge, this case presents with more extensive bleeding and cystic degeneration than any of the previous reported cases. In our case, due to the large range of cystic lesions and hemorrhage, the mass was slightly hyperintense on T1-weighted imaging and had high-low mixed signal intensity on T2-weighted imaging with ring-like and septal enhancement.

The clinical symptoms of renal schwannoma are nonspecific; a small number of patients do not have any symptoms, and the mass is only found incidentally during physical examination for any number of reasons. Among the 37 previous cases, the most common symptoms were abdominal pain (51.4%, mostly flank pain), hematuria (21.6%) and fever (16.2%). Other symptoms included nausea, vomiting, loss of appetite, and weight loss. In our report, the patient exhibited persistent flank pain. As the tumor grew, it pressed on the surrounding organs and tissues, resulting in pain that worsened and caused dyspnea during emotional agitation and when lying in the supine position. In addition, renal schwannomas grow slowly. When the tumor grows to a certain volume, the abdominal mass can be palpated.

Radical nephrectomy or partial nephrectomy are recommended as first-line treatments for renal schwannomas. Histologically, a typical schwannoma consists of Antoni A and Antoni B tissue. Antoni A tissue is composed of spindle cells arranged in a palisade with Verocay bodies, while Antoni B tissue is composed of loose and scattered cells with many myxoid changes [33]. S-100 protein immunostaining was positive in all cases.

In conclusion, renal schwannomas are rare and grow slowly. Cystic degeneration in the tumor is a common imaging feature. When a middle aged-elderly patient has a well-defined renal tumor with obvious cystic degeneration and shows mild to moderate homogeneous or heterogeneous enhancement, renal schwannoma should be considered. However, pathological examination is the gold standard for diagnosis. We report a large renal schwannoma with obvious hemorrhage and cystic degeneration, which could be used as a reference for further study.

## Data Availability

The datasets used and analysed during the current study available from the corresponding author on reasonable request.

## Abbreviations

CT: Computed tomography
MRI: Magnetic resonance imaging
US: Ultrasound
